# Enhancement of carotenoid biosynthesis in the green microalga *Dunaliella salina* with light-emitting diodes and adaptive laboratory evolution

**DOI:** 10.1007/s00253-012-4502-5

**Published:** 2012-10-25

**Authors:** Weiqi Fu, Ólafur Guðmundsson, Giuseppe Paglia, Gísli Herjólfsson, Ólafur S. Andrésson, Bernhard Ø. Palsson, Sigurður Brynjólfsson

**Affiliations:** 1 Center for Systems Biology, University of Iceland, 101 Reykjavík, Iceland; 2 Department of Bioengineering, University of California, San Diego, La Jolla, CA 92093-0412 USA

**Keywords:** *Dunaliella salina*, Adaptive laboratory evolution, β-carotene and lutein, Carotenoid metabolism, LED-based photobioreactor

## Abstract

There is a particularly high interest to derive carotenoids such as β-carotene and lutein from higher plants and algae for the global market. It is well known that β-carotene can be overproduced in the green microalga *Dunaliella salina* in response to stressful light conditions. However, little is known about the effects of light quality on carotenoid metabolism, e.g., narrow spectrum red light. In this study, we present UPLC-UV-MS data from *D. salina* consistent with the pathway proposed for carotenoid metabolism in the green microalga *Chlamydomonas reinhardtii*. We have studied the effect of red light-emitting diode (LED) lighting on growth rate and biomass yield and identified the optimal photon flux for *D. salina* growth. We found that the major carotenoids changed in parallel to the chlorophyll *b* content and that red light photon stress alone at high level was not capable of upregulating carotenoid accumulation presumably due to serious photodamage. We have found that combining red LED (75 %) with blue LED (25 %) allowed growth at a higher total photon flux. Additional blue light instead of red light led to increased β-carotene and lutein accumulation, and the application of long-term iterative stress (adaptive laboratory evolution) yielded strains of *D. salina* with increased accumulation of carotenoids under combined blue and red light.

**Electronic supplementary material:**

The online version of this article (doi:10.1007/s00253-012-4502-5) contains supplementary material, which is available to authorized users.

## Introduction

Microalgae have great potential in many aspects of the conversion of conventional petrol-based manufacturing to bio-based manufacturing: in production of biofuels as well as bio-factories producing valuable pharmaceuticals, food additives, and cosmetics (Cordero et al. 2011; Lamers et al. 2008; Takaichi 2011; Vilchez et al. 2011; Wijffels and Barbosa 2010). Carotenoids are extremely important for human and animal nutrition, and they are distributed broadly in both phototrophic and non-phototrophic organisms (Takaichi 2011). However, humans and animals cannot synthesize necessary carotenoids and must obtain them from their diets (Takaichi 2011). Carotenoids can be divided into two groups based on their chemical structure: the carotenes such as β-carotene and the xanthophylls such as lutein. Among important carotenoids for humans, β-carotene is a major source of vitamin A which is necessary for functions of the retina and has an effect on many tissue types (Amengual et al. 2011; von Lintig et al. 2005) through its action as a regulator of gene expression. In addition, β-carotene helps protect the skin against photoaging by its antioxidant activity (Darvin et al. 2011). Lutein and zeaxanthin are also of particular interest for their role in reducing the development and progression of age-related macular degeneration (Carpentier et al. 2009; Fernandez-Sevilla et al. 2010).

In higher plants as well as in green algae, the antenna pigment molecules (Telfer 2002) bound to light harvesting (or antenna) complexes in the thylakoid membrane help to harvest light and transfer energy to the reaction center of photosystems, e.g., PS II. The antenna pigments usually consist of carotenoids, chlorophyll *b*, and chlorophyll *a* (Jahns and Holzwarth 2012; Telfer 2002). Chlorophyll *a* is very different from chlorophyll *b* in functions since it acts uniquely as primary electron donor in the reaction center of photosystems, though it also helps to transfer energy in the antenna complex (Jahns and Holzwarth 2012). Carotenoids such as β-carotene and lutein (Jahns and Holzwarth 2012; Telfer 2002) play a central role in PS II, harvesting blue light and transferring energy to photosystem reaction centers and protecting the photosynthetic apparatus against photo-oxidative damage by deactivating reactive oxygen species (ROS) and reducing the ROS formation under excess light. To study carotenoid metabolism in the green microalga *Dunaliella salina*, it will be informative and important to profile all related antenna pigments.

The unicellular green microalga *D. salina* has been useful in studying carotenoid metabolism as it is able to accumulate large amounts of carotenoids (Ye et al. 2008). To date, some researchers have addressed the effects of different abiotic environment conditions (Gómez and González 2005; Coesel et al. 2008; Lamers et al. 2010; Ramakrishna et al. 2011) on the accumulation of carotenoids in *D. salina*, and it is widely accepted that light intensity is a key stimulus for β-carotene overproduction in *D. salina* (Lamers et al. 2010). With regard to the regulation of genes involved in carotenoid biosynthesis in *D. salina*, it has also been suggested that *Lcy-β* steady-state transcript levels were upregulated in response to all stress conditions tested, e.g., salt, light, and nutrient depletion (Ramos et al. 2008). However, little is known about the light regulation underlying carotenoid metabolism, and it remains unclear whether accumulation of β-carotene and major carotenoids is related to light quality, although an increase in β-carotene accumulation has been observed in *Dunaliella* cultivated under white light combined with UV-A, compared with white light alone (Salguero et al. 2005; Lamers et al. 2008; Mogedas et al. 2009). The progressive light-emitting diode (LED) technology that is currently emerging has a high conversion efficiency from electricity to light while providing narrow spectra of wavelengths, and the application of LED in photobioreactors (PBRs) marks a great advance over existing indoor agricultural lighting (Yeh and Chung 2009). In addition, LED illumination induced light stress on *Dunaliella* cells at lower incident photon fluxes, e.g., 170 and 255 μE/m^2^/s, while as high as 1000 μE/m^2^/s photon flux is usually provided for driving *Dunaliella* cells to overproduce β-carotene by conventional lights such as fluorescent lamp and high-pressure sodium lamp (Lamers et al. 2010). In this study, the effects of nearly monochromatic light (20 nm bandwidth at half peak height), e.g., red light with a narrow spectrum as well as combined blue and red light, on *D. salina* were evaluated, with regard to both growth rate and the accumulation and composition of major carotenoids.

Adaptive laboratory evolution (ALE) has been widely utilized as a tool for developing new biological and phenotypic functions and exploring strain improvement in synthetic biology for bacteria (Palsson 2011). Specifically, ALE has been utilized to evolve strains to better adapt to defined conditions, e.g., a certain carbon source, energy source, or to cope with environmental stress. However, ALE is still a novel solution for improving strain performance in microalgal biotechnology (Fu et al. 2012). With the aid of redesigned LED-based PBRs combining blue LED with red LED, we have set out to use ALE to develop *D. salina* strains with increased yields of carotenoids.

## Materials and methods

### Microalga and growth conditions

*D. salina* was purchased from the University of Texas (UTEX LB #200) and grown in Gg-8 medium [see Table S1 in the Electronic supplementary material (ESM)] by modifying Gg medium (Jahnke 1999) at 25 ± 2 °C. Culture pH was regulated between 6.5 and 7.5, unless otherwise indicated. Seed culture of *D. salina* cells was grown under lower light intensity (85 μE/m^2^/s) of red LED lighting till late exponential phase and then used in different experiments. In all experiments, *D. salina* was grown for 5 days, unless otherwise indicated. The initial biomass concentration for all experiments was relatively high (*A*_660nm_ > 1.0), and it was assumed that the supplied light measured on the inner surface of PBR was all absorbed by *D. salina* cells during batch culture.

### Parameters for photobioreactors

Bubble column photobioreactors (PBRs) were cylindrical with *H* = 30 cm, *D* = 4 cm, and a working volume of 300 ± 5 ml (Fu et al. 2012). Input gas was 90 ml/min of 2.5 % CO_2_ in air.

### Artificial light supply and setup

Although in the green microalga *Chlamydomonas reinhardtii* (Merchant et al. 2007) it has been calculated that red LED light centered at 674 nm yields the maximum photon utilization efficiency for growth (Chang et al. 2011), the most efficient and inexpensive red LED, based on the (Al, Ga) InP system, emits light centered at a shorter wavelength, or 660 nm (Krames et al. 2007). Red (Part number: SSL-LX5093SRC) and blue (Part number: VAOL-5LSBY2) LED arrays with a narrow output spectrum (20 nm bandwidth at half peak height) centered at 660 and 470 nm, respectively, were then purchased from LUMEX Inc. (Taiwan, China). The intensity of light supplied to the PBR was measured using a quantum sensor (SR. NO. Q40526 of QUANTUM, Model LI-1400, LI-COR biosciences, Lincoln, NE, USA) on the inner surface of each PBR. Average light intensity was varied by using different duty cycles at the same frequency (10 kHz) of flashing light (Park and Lee 2000; Fu et al. 2012). For example, a 15 % duty cycle with a 10-kHz frequency means that the light was on for 15 % of the duration of one on/off cycle (0.1 ms).

### Adaptive laboratory evolution with blue light stress

ALE (Palsson 2011) was conducted by means of a semi-continuous culture system with repeated 5-day cycle. For starting each new cycle, the culture was diluted to the same cell density (approximately 0.5 gDCW/L) by removing part of the culture and refilling same volume of fresh medium. Combined blue and red LED light (microarrays, 1:3) was supplied for PBR at a total photon flux of 170 μE/m^2^/s.

### Biomass determination

Samples of cell suspension (usually 5 ml) were collected on a mixed cellulose membrane (pore size, 0.45 μm), washed twice with deionized water, and dried for 24 h at 60 °C before weighting.

### Biomass yield relative to light energy

The biomass yield in relation to light (the quantum yield) *ψ*_*E*_ (Molina-Grima et al. 1997) is defined as the amount of biomass generated per unit of radiation absorbed by the algal culture. The equation for calculating biomass yield on light was expressed as below (Fu et al. 2012):

1 $$ {\psi_E}=\frac{{{P_{\mathrm{b}}}}}{{{F_{\mathrm{vol}}}}} $$

where ${P_{\mathrm{b}}}$ stands for the volumetric biomass productivity and ${F_{\mathrm{vol}}}$ for the incident photon flux absorbed per unit volume. ${F_{\mathrm{vol}}}$ was calculated by the following equation:

2 $$ {F_{\mathrm{vol}}}=I{}_0\times {S_0}/{V_0} $$

where *I*_0_ stands for the incident light intensity on the surface of the culture, *V*_0_ for the working volume, and *S*_0_ for the surface area of culture.

### Chlorophyll and carotenoid analysis

An aliquot of 0.5-ml cell suspension was centrifuged at 1,000×*g* for 10 min. The cell pellet was then extracted with 3 ml of ethanol: hexane 2:1 (*v/v*) containing 0.1 % (*w/v*) butylated hydroxytoluene till colorless (Garcia-Gonzalez et al. 2005; Gentili and Caretti 2011). Two milliliters of water and 4 ml of hexane were added, and the mixture was vigorously shaken and centrifuged again at 1,000×*g* for 5 min. Four milliliters of the upper hexane layer was evaporated under N_2_ at 25 ± 2 °C, reconstituted in methyl tertiary butyl ether : acetonitrile (MTBE/CAN; 50:50), and analyzed by ultra-performance liquid chromatography, UV and mass spectrometry detection (UPLC-UV-MS).

UPLC separation was performed on an ACQUITY UPLC (Waters, MS technologies, UK) by reversed phase chromatography using an ACQUITY UPLC HSS T3 1.8 μm column (2.1 × 150 mm; Waters, Manchester, UK). The mobile phase consisted of phase A, a mixture of ACN/methanol/MTBE (70:20:10, *v*/*v*/*v*), and phase B, 10 mM ammonium acetate. The elution flow rate was 0.45 ml/min with a gradient of 60 % phase A at 0 min, 75 % at 5 min, 100 % at 17.5 min, 98 % at 24 min, and 60 % between 25 and 30 min. A TUV detector (Waters, MS technologies, UK) was used for UV detection at 450 nm.

The inlet system (UPLC-UV system) was coupled in line with a quadrupole-time of flight hybrid mass spectrometer (Synapt G2, Waters, Manchester, UK), using electrospray ionization interface (positive mode) to direct column eluent to the mass spectrometer. The mass spectrometer was operated in V mode for high sensitivity using a capillary voltage of 3 kV and a cone voltage of 30 V. Cone and desolvation gas flow were 20 and 800 L/h, respectively, while source and desolvation gas temperature were 100 and 500 °C, respectively. Leucine enkephalin (2 ng/μL) was purchased from Sigma-Aldrich, Co. LLC (St. Louis, MO, USA) and used as lock mass (*m/z* 556.2771). MarkerLynx (Waters, Manchester, UK) was used to integrate and align MS data points and to convert them into exact mass retention time pairs. QuanLynx (Waters, Manchester, UK) was used to integrate chromatograms for quantitative analysis. Metabolites identification was achieved using retention time, UV detection, and exact mass measurement (Δppm < 10). Further confirmation was provided by data-dependent MS/MS experiments for structural elucidation.

ACN was purchased from Merck (Darmstadt, Germany). Water was obtained by using an 18-Ωm Milli-Q (Millipore, USA). All chemicals and solvents were of analytical grade or higher purity. For quantitative analysis, standard chlorophyll *a*, chlorophyll *b*, lycopene, β-carotene, lutein, and zeaxanthin were purchased from Sigma-Aldrich, Co. LLC (St. Louis, MO, USA). Specifically, MS detection was used to indentify untargeted pigment compounds in *D. salina* cell samples, and standards were used to make calibration curves for quantification of pigments by UPLC under UV detection conditions. In addition, violaxanthin, antheraxanthin, and zeaxanthin (VAZ) pool was calculated by normalizing the intensity (area) of signals detected by UV detector for violaxanthin, antheraxanthin, and zeaxanthin in total to zeaxanthin for which standard zeaxanthin was used for quantification.

## Results

### Analysis of major antenna pigments

**Fig. 1:**
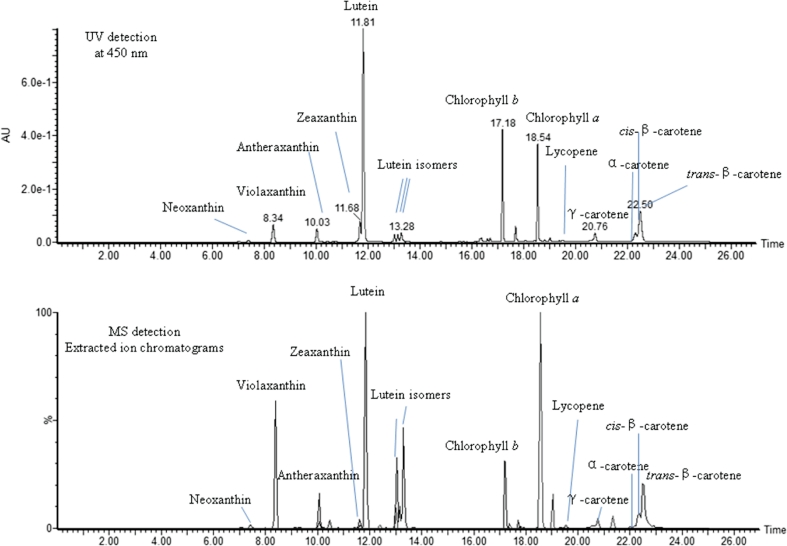
Profile of major pigments in *D. salina* detected by UPLC-UV-MS (LC/MS). The cells were cultivated for 5 days under red LED lighting at 85 μE/m^2^/s. Extracted ion chromatograms for the carotenoids species in MS detection figure were scaled up by six times. Operation conditions of UPLC-UV-MS were described in the “Materials and methods” section

**Fig. 2:**
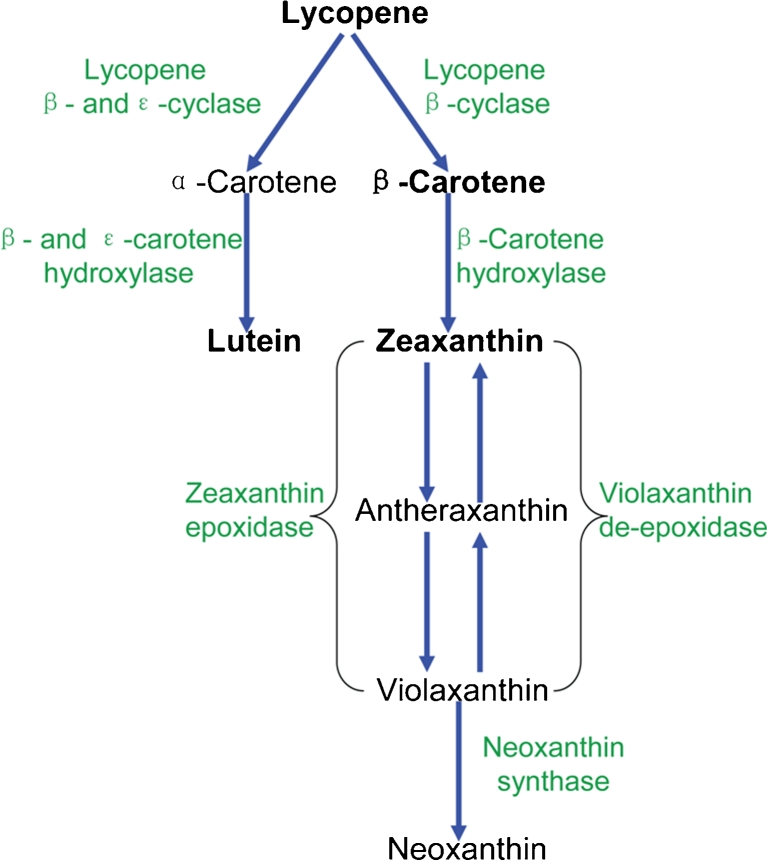
Proposed pathway of carotenoid metabolism in *D. salina* based on *C. reinhardtii* in KEGG database

With the aid of UPLC-UV-MS, we were able to quantify targeted pigments and identify unexpected compounds in *Dunaliella* samples (see details in “Materials and methods” section). We have detected the same major carotenoids and chlorophylls (Fig. 1) as found in *C. reinhardtii* as shown in the KEGG database (Kanehisa et al. 2012). These results support the notion (Fig. 2) that *D. salina* has the same major features of caratenoid metabolism as has been found in other chlorophyte algae shown in KEGG database (Kanehisa et al. 2012).

### *D. salina* growth under red LED lighting

**Fig. 3:**
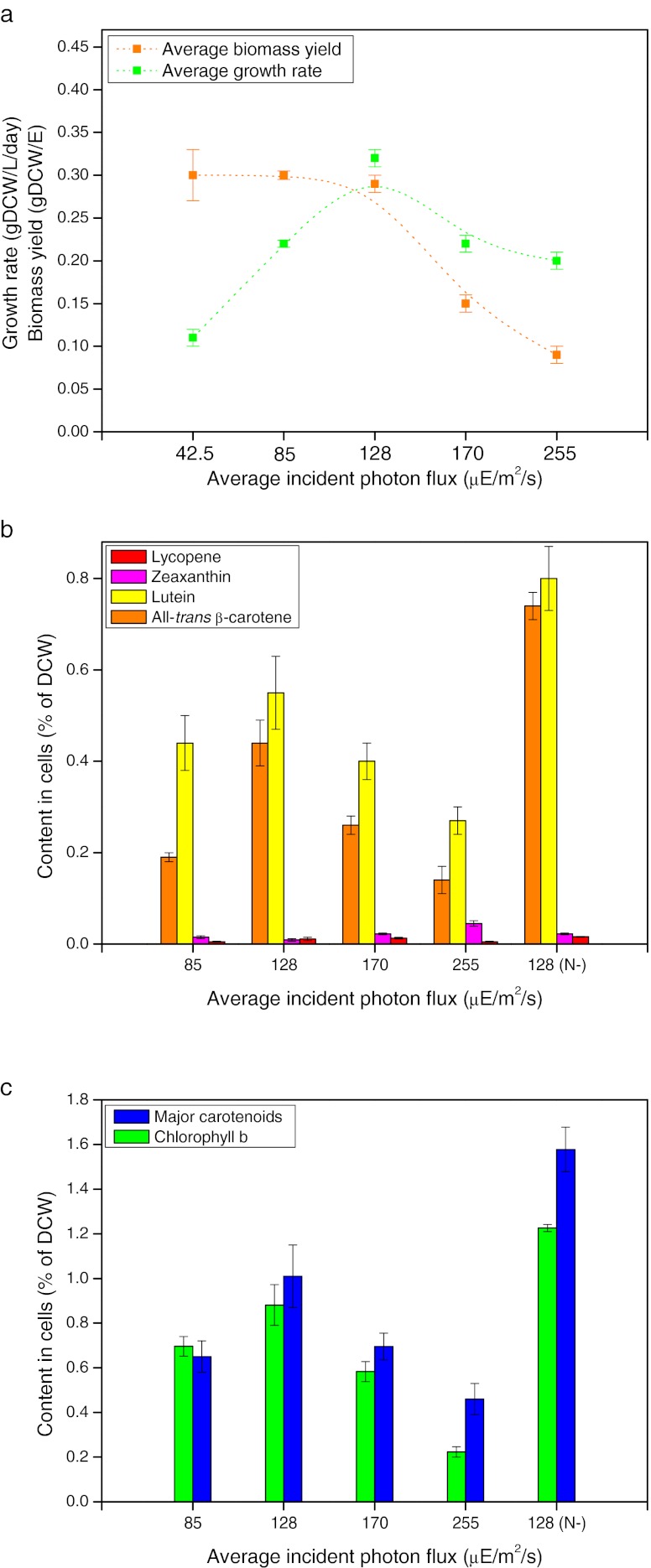
*D. salina* growth and carotenoid accumulation under red LED lighting. Cells were cultivated for 5 days under different light intensity conditions, while cells of 128 (N-) were cultivated for additional 16 days for nitrogen deprivation. Detailed data were presented in Table S2 in the ESM. **a** Average growth rate and biomass yield on light energy under varied light intensities. Average growth rate or biomass productivity indicated biomass produced per day during batch culture, and average biomass yield was calculated according to the average growth rate (see Fig. S2 in the ESM). *Dotted lines* are drawn to guide the eye. **b** Lycopene, all-*trans* β-carotene, lutein, and zeaxanthin content. **c** Chlorophyll *b* and major carotenoids content. All results are averaged from three independent experiments. *Error bars* indicate SD

We have chosen a LED-based PBR system to grow *D. salina* (Fig. 3; see “Materials and methods” section). In our experiments, *D. salina* was cultivated at a high CO_2_ level of 2.5 % (*v/v*) with a gas flow rate of 90 cm^3^/min (corresponding to a superficial velocity of 0.12 cm/s, calculated in the conventional way by dividing the gas flow rate with the cross-sectional area of the PBR). Gg-8 medium (see details in Table S1 in the ESM) was designed to support a biomass capacity of 5 gDCW/L (Fig. S1 in the ESM). Therefore, in this study, growth of *D. salina* below a concentration of 5 gDCW/L was not constrained by lack of nutrients in the medium.

The growth profiles of *D. salina* under different light intensities could be divided into two groups, light-dependent growth (approximately linear) and light-inhibited growth (Fig. 3a and Fig. S2 in the ESM). Specifically, growth of *D. salina* was either light-dependent with a photon flux of 128 μE/m^2^/s or lower (85 and 42 μE/m^2^/s), or light-inhibited when the incident photon flux was 170 or 255 μE/m^2^/s. Further, the maximum average growth rate was 0.32 gDCW/L/day at a photon flux of 128 μE/m^2^/s, and the average biomass yield was similar under all light-dependent conditions. As the growth rate increased in a linear fashion over the range of 42 to 128 μE/m^2^/s, it appears that the latter photon flux is suitable for efficient growth of *D. salina*.

### Carotenoid content under different growth conditions

In this section, the effects of different light intensities on carotenoid production were evaluated. We have found that all-*trans* β-carotene and lutein are major pigments in *D. salina* cells in addition to chlorophylls (Figs. 1 and 3). The content of all-*trans* β-carotene, lutein, lycopene (the precursor of both β-carotene and lutein), and zeaxanthin (the first downstream metabolite of β-carotene) was further analyzed under varied intensities of red LED light over a range of 85 to 255 μE/m^2^/s (Fig. 3a). The major carotenoids (Fig. 3c), primarily all-*trans* β-carotene and lutein (Fig. 3b), increased as the photon flux increased from 85 to 128 μE/m^2^/s and then decreased with additional photon flux under light-inhibiting conditions (Fig. 3 and Fig. S2 in the ESM). As previously documented (Coesel et al. 2008; Lamers et al. 2008), nitrogen starvation enhanced the accumulation of major carotenoids in *D. salina* (Figs. 3b, c). We also found that chlorophyll *b* content as well as chlorophyll *a* (Table S2 in the ESM) changed in accordance with major carotenoids in *D. salina* (Fig. 3c), and the ratio of major carotenoids to chlorophyll *b* increased with growing light intensity.

### Effect of combined blue and red LED lighting on *D. salina* growth

High intensity of red light did not yield more carotenoids in cells, presumably due to serious photodamaging and bleaching (Fig. 3a). We tried to adapt *D. salina* to grow at 170 μE/m^2^/s of red LED light using semi-continuous culture, but the cells were sensitive to red light at this level, were damaged significantly, and failed to recover after iterative red light stress.

Since increasing the photon flux of red LED light from 128 to 170 μE/m^2^/s (Fig. 3) decreased both growth rate and carotenoid accumulation in *D. salina*, we attempted to improve production efficiency by adding blue LED (peak at 470 nm) to the red LED (peak at 660 nm) with a total incident photon flux of 170 μE/m^2^/s in which red light and blue light were 128 and 42 μE/m^2^/s, respectively. The *Dunaliella* growth under combined blue and red LED lighting exhibited an average growth rate of 0.40 ± 0.01 gDCW/L/day and biomass yield of 0.27 ± 0.01 gDCW/E. For comparison, the values were 0.22 ± 0.01 gDCW/L/day and 0.15 ± 0.01 gDCW/E, respectively under 170 μE/m^2^/s (photoinhibiting) red LED lighting (Table S3 in the ESM). These results indicated that the growth was not significantly inhibited by addition of 42 μE/m^2^/s blue light to 128 μE/m^2^/s red light in contrast to the same addition of red light.

### *Dunaliella* strain development using adaptive laboratory evolution

**Fig. 4:**
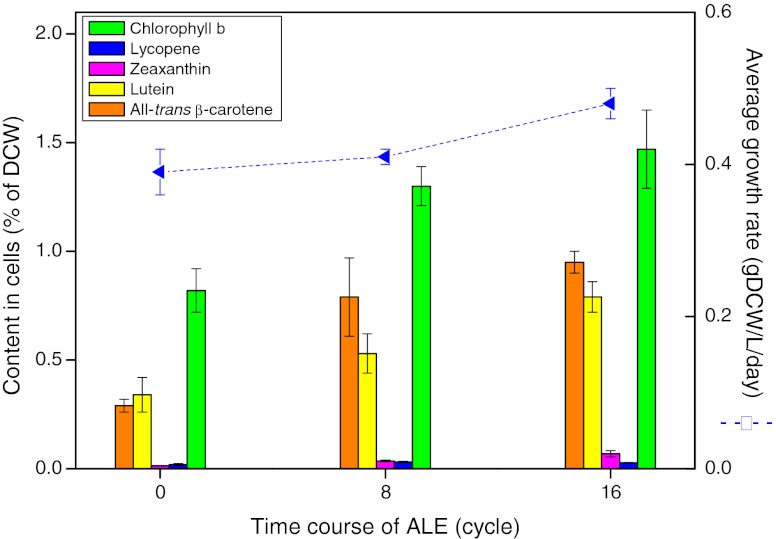
Effect of adaptive laboratory evolution (ALE) on growth rate and carotenoid accumulation in *D. salina*. Average growth rate indicated biomass produced per day during one cycle. All the cycles including cycle 0 were performed under a total light intensity of 170 μE/m^2^/s consisting of 42 μE/m^2^/s blue LED light and 128 μE/m^2^/s red LED light. Detailed data were presented in Table S2 in the ESM. The results are averaged from three independent experiments. *Error bars* indicate SD

We started ALE with redesigned LED lighting at a total photon flux of 170 μE/m^2^/s. Since *D. salina* growth under red LED lighting was light-dependent at 128 μE/m^2^/s and light-inhibited at 170 μE/m^2^/s, respectively (Fig. 3a), we reasoned that 42 μE/m^2^/s of blue light might induce light stress on cells without damaging cells. Indeed, increased stress was indicated by an increased level of zeaxanthin relative to members of the xanthophyll cycle (Fig. 2 and Fig. S3 in the ESM) since the amount of zeaxanthin synthesized via the xanthophyll cycle is highly correlated with the level of energy-dependent quenching in most plants (Jahns and Holzwarth 2012; Müller et al. 2001). After 16 cycles of ALE, the all-*trans* β-carotene and lutein content was increased to 3.3 times and 2.3 times of initial levels, respectively, while the average growth rate had increased 20 % (Fig. 4). Interestingly, the adapted *D. salina* cells (HI001) exhibited enhanced light tolerance under red LED lighting at a total photon flux of 170 μE/m^2^/s and gained an average growth rate of 0.40 ± 0.01 gDCW/L/day and biomass yield of 0.27 ± 0.01 gDCW/E, approximately 1.8 times of original levels. Effects of ALE were evaluated further by comparisons between original *D. salina* cells and the adapted equivalents (Table 1). Both average growth rate and the content of major carotenoids increased by ALE under the condition of blue (42 μE/m^2^/s) and red (128 μE/m^2^/s) LED lighting, but the adapted strains (HI 001) and the original *D. salina* (UTEX LB #200) had very similar performance under 128 μE/m^2^/s red LED light. Further, the major pigment compositions (chlorophyll *a*, chlorophyll *b*, and major carotenoids; Table 1) were very similar for adapted strains (HI 001) and the original *D. salina* (UTEX LB #200) under the usual culture conditions, i.e., 128 μE/m^2^/s red LED light. Thus, these traits accumulated by ALE were condition-specific, dependent on providing blue light in addition to nearly saturating red light.

**Table 1:** Effects of ALE on *D. salina* growth and carotenoid metabolism

Light sources	Light intensity (μE/m^2^/s)	Average growth rate (gDCW/L/day)	Average biomass yield (gDCW/E)	Changes of carotenoids content^a^	Changes of chlorophyll *b* content^b^	Changes of chlorophyll *a* content^c^	*D. salina* ^d^
Red LED	128	0.32 ± 0.01	0.29 ± 0.01	100 %	100 %	100 %	UTEX LB #200
		0.33 ± 0.02	0.30 ± 0.01	96.1 ± 3.5 %	88.3 ± 4.1 %	102.9 ± 6.0 %	HI 001
Blue and red LED (1:3)	170	0.40 ± 0.01	0.27 ± 0.01	100 %	100 %	100 %	UTEX LB #200
		0.48 ± 0.02	0.33 ± 0.01	285.1 ± 55.3 %	181.5 ± 30.5 %	202.9 ± 21.0 %	HI 001

Growth data were averaged from three independent experiments of batch culture (means ± SD)

^a^Carotenoids contents in original cells (UTEX LB #200) were set as references (100 %) for both light conditions separately. Carotenoids content refers to a sum content of four carotenoids, namely, lycopene, all-*trans* β-carotene, lutein, and zeaxanthin.

^b^Chlorophyll *b* contents in original cells (UTEX LB #200) were set as references (100 %) for both light conditions separately

^c^Chlorophyll *a* contents in original cells (UTEX LB #200) were set as references (100 %) for both light conditions separately

^d^ *D. salina* cells without ALE treatment were original species (UTEX LB #200), while the *D. salina* cells after experiencing consecutive ALE treatment were regarded as adapted species (named as HI 001)

## Discussion

**Fig. 5:**
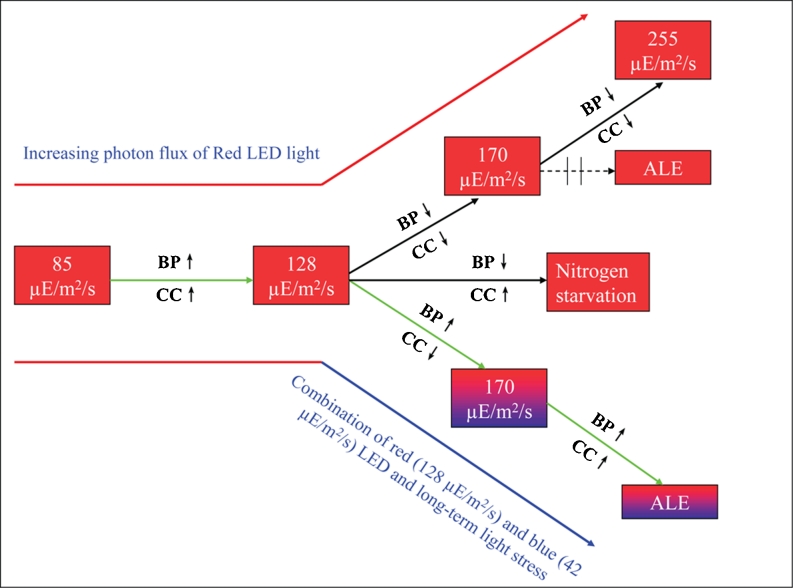
Schematic pathways to developing *D. salina* with increased yields of carotenoids. *BP* biomass productivity, *CC* carotenoids content

Global climate change has called for immediate reduction of CO_2_ emission and development of sustainable manufacturing. This study has provided data relating to such an issue, the photosynthesis-based production of valuable compounds such as β-carotene and lutein using microalgae. The use of well-designed LED lighting for *D. salina* illustrates the potential for enhancing sustainable production of carotenoid products such as β-carotene and lutein efficiently by microalgal biotechnology (Lamers et al. 2008; Ribeiro et al. 2011). Major results of this study are summarized in Fig. 5. Firstly, the metabolic profile of major pigments was determined by UPLC-UV-MS and found to be consistent with a model of carotenoid metabolism proposed for green algae (Chang et al. 2011). Then, we studied the effect of red LED lighting on the average growth rate and photon based biomass yield. The photon flux of 128 μE/m^2^/s red light (660 nm) was determined optimal for efficient growth of *D. salina*. The major antenna pigments in *D. salina* were analyzed under different red LED lighting conditions, and it was inferred from the results that the stress due to supra-optimal photon flux of narrow bandwidth red light did not yield higher carotenoid levels in *D. salina* presumably due to significant photodamage. The major carotenoids increased relative to chlorophyll *b* at high light intensities. Finally, using redesigned lighting combining red LED (75 %) arrays with blue LED (25 %) arrays, we were able to utilize adaptive laboratory evolution (ALE) to develop strains yielding higher levels of carotenoids.

In plant cells, mechanisms regulating carotenoid biosynthesis and accumulation are complex (Lu and Li 2008). It has been suggested that light plays a key role in the biosynthesis of carotenoids through light signal sensing and downstream regulation (Lamers et al. 2008). However, it is difficult to study light effects on *Dunaliella* cells in depth without high-quality lighting. With well-designed LED lighting, it has become possible to investigate effects of nearly monochromatic light, e.g., red light and blue light with narrow spectra, on both *Dunaliella* cell growth and carotenoid metabolism. Our results demonstrate that *Dunaliella* cells (UTEX LB #200) are sensitive to a red LED photon flux of 170 μE/m^2^/s and higher applied in this study and fail to acclimate to such environments. Further analysis of the ratio of zeaxanthin to the VAZ pool (Fig. S3 in the ESM) is indicative of stress responses to high photon flux supporting the observation that with a basic flux of 128 μE/m^2^/s red LED light, additional red LED light is more stressful than additional blue LED light. The light signal transduction of blue light may be different from that of red light since plants usually have different photoreceptors/domains, e.g., blue light-regulated and red light-regulated, although these photoreceptors have both overlapping and distinct functions (Chory 2010). It has also been found that blue LED light enhanced growth of the green microalga *Haematococcus pluvialis* in the early exponential phase but caused the suppression of growth later in batch culture, while accumulation of astaxanthin was significantly enhanced (Katsuda et al. 2004). In addition, it has been reported that blue light stimulates carotenoid synthesis in non-photosynthetic bacteria such as *Myxococcus xanthus* (Ruiz-Vázquez et al. 1993). A possible mechanism may be that blue light signal transduction in *D. salina* involving major carotenoids (Chory 2010; Jahns and Holzwarth 2012) is separate from red light at a high level of total light intensity. It has been shown that adaptation to environmental factors varies along native clines, and it has been suggested that changes in photoreceptor family members are important determinants in adaptation to the natural variation of light sensitivity (Chory 2010). The stable difference in the adapted strain (HI 001) could be either a consequence of accumulative mutations or due to selection of variants already found in the original culture (UTEX LB #200). Further study, e.g., by reference genome sequencing, needs to be performed to decipher the nature of the differences in the *Dunaliella* strains (both UTEX LB #200 and HI 001) once the genome sequences of *D. salina* strains UTEX 1644 and CCAP 19/18 are published by the US DOE Joint Genome Institute (http://genome.jgi.doe.gov/genome-projects/).

Biosynthesis of carotenoids is complex and coordinated with the biogenesis of chlorophylls and proteins of the photosynthetic apparatus (Bohne and Linden 2002) as well as electron transport (Cardol et al. 2011). The content of the major carotenoids appears to be regulated in concert with the chlorophyll *b* content in *D. salina* cells (Figs. 3c and 4). It is possible that carotenoid metabolism is regulated along with chlorophyll *b* through the geranyl geranyl diphosphate pathway (KEGG database) as summarized in Fig. S4 in the ESM. Although supra-optimal irradiation with red light did not increase carotenoids but seriously inhibited growth, we found that adding excess blue light, and applying ALE on the contrary, led to increased β-carotene and lutein yields. Our results show that well-designed ALE is an effective way to increase sustained productivity in contrast to established methods where carotenoid accumulation in *D. salina* is usually achieved with low biomass productivity and cell density. We have shown that an efficient culture system with increased light energy efficiency and economy of operation can be developed using innovation in lighting technology in combination with genetically based methods such as ALE for strain development.

In conclusion, light quality is critical for both *D. salina* growth and carotenoid accumulation. ALE combined with redesigned LED lighting has allowed a substantial increase in growth yield per photon flux and in the level of sustainable production of β-carotene and lutein (Fig. 4). These results are also a demonstration of the technical feasibility of LED-based PBRs for direct conversion of CO_2_ to valuable chemicals.

## Electronic supplementary material

Below is the link to the electronic supplementary material.

ESM 1 (PDF 95 kb)
