# Supplementary material for: Enhancement of carotenoid biosynthesis in the green microalga Dunaliella salina with light-emitting diodes and adaptive laboratory evolution
Source: Appl Microbiol Biotechnol. 2012 Oct 25;97(6):2395–403. doi: 10.1007/s00253-012-4502-5 (PMC3586100; doi:10.1007/s00253-012-4502-5)
Supplement: Supplementary file 1 — (PDF 95 kb) [file 253_2012_4502_MOESM1_ESM.pdf]

Title:

Enhancement of carotenoid biosynthesis in the green microalga *Dunaliella salina* with light-emitting diodes and adaptive laboratory evolution

Author affiliations:

Weiqi Fu[1], Ólafur Guðmundsson[1], Giuseppe Paglia[1], Gísli Herjólfsson[1], Ólafur S. Andrésson[1*], Bernhard Ø. Palsson[1,2], Sigurður Brynjólfsson[1*]

[1]Center for Systems Biology, University of Iceland, 101 Reykjavík, Iceland. [2]Department of Bioengineering, University of California, San Diego, La Jolla, CA 92093–0412, U.S.A.

Corresponding author:

* Correspondence should be addressed to Ólafur S. Andrésson (osa@hi.is);

or Sigurður Brynjólfsson (sb@hi.is), Tel: +354-525-4641, fax: +354-525-4632.

Supplementary figures and tables

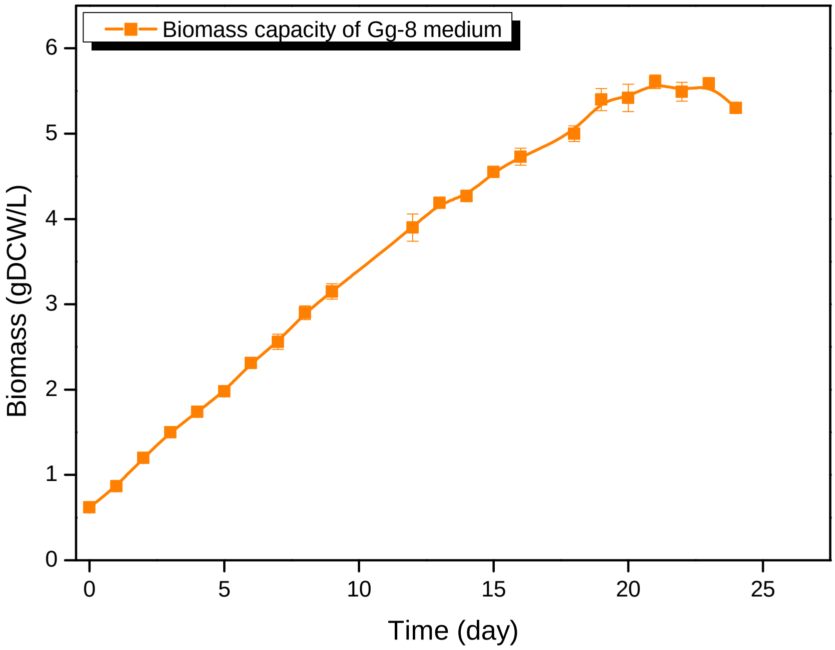

Fig. S1 Biomass capacity of Gg-8 medium for cultivating *D. salina*. Incident average photon flux of red LED light was 85 µE/m^2/s. The results presented are values averaged from three independent experiments. Error bars indicate SD.

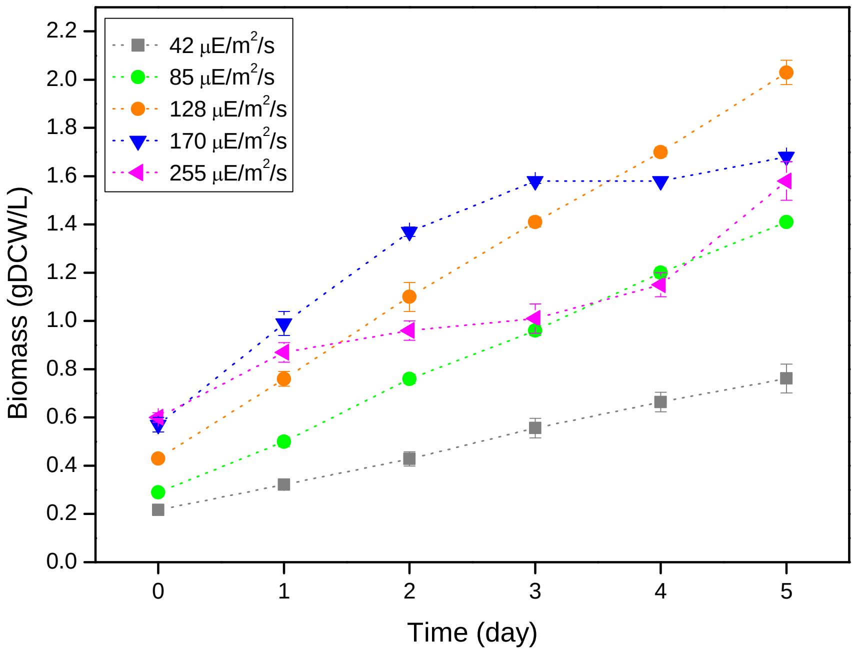

Fig. S2 Growth of *D. salina* under red LED lighting. Average photon flux was controlled by varying the duty cycles. Initial biomass concentration was increased along with the increase in light intensity at low level (42, 85 and 128 μE/m^2/s) and kept the same at high level of light intensity (170 and 255 μE/m^2/s). The results presented are values averaged from three independent experiments. Dotted lines are drawn to guide the eye. Error bars indicate SD.

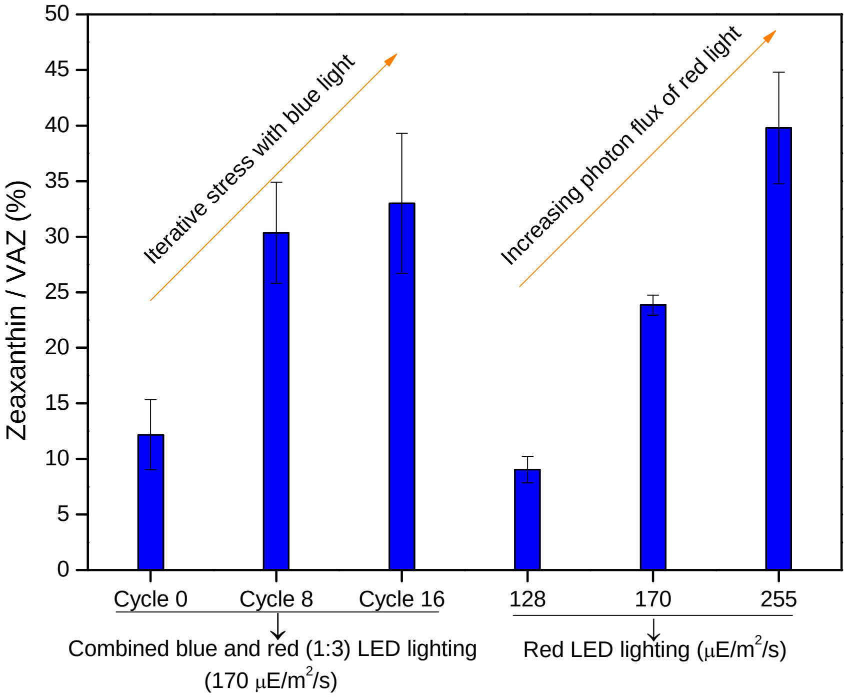

Fig. S3 Effects of combined blue and red LED lighting and increasing red LED lighting on zeaxanthin percentage in VAZ (violaxanthin, antheraxanthin, and zeaxanthin) pool (xanthophyll cycle as shown in Fig. 2). All the ALE cycles including cycle 0 were performed under a total light intensity of 170 $\mu E/m^2/s$ consisting of 42 $\mu E/m^2/s$ blue LED light and 128 $\mu E/m^2/s$ red LED light. The VAZ pool was calculated by normalizing the intensity of signals detected by UV detector for violaxanthin, antheraxanthin, and zeaxanthin pool to zeaxanthin (see Materials and Methods). The results are averaged from three independent experiments. Error bars indicate SD.

The linker between carotenoid and chlorophyll metabolism

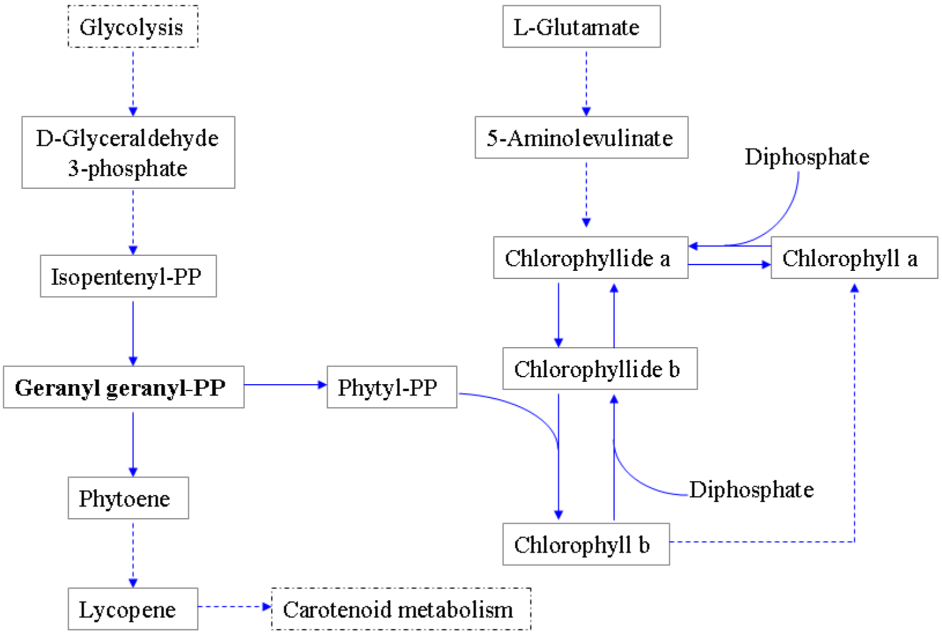

Fig. S4 Proposed link between carotenoid and chlorophyll metabolism in *D. salina* based on model pathway of *Chlamydomonas reinhardtii* (Chang et al. 2011) shown in KEGG database (Kanehisa et al. 2012).

Table S1 Nutrient composition of Gg-8 medium.

Compound	Molar concentration
NaCl	1.5 M
KNO_3	31.2 mM
$MgSO_4 \cdot 7H_2O$	5.2 mM
H_3BO_3	4.0 mM
$Na_2HPO_4 \cdot 2H_2O$	0.75 mM
$CaCl_2 \cdot 2H_2O$	0.085 mM
FeNaEDTA	27.2 μM
$ZnSO_4 \cdot 7H_2O$	16.0 μM
$MnCl_2 \cdot 4H_2O$	3.5 μM
$CuSO_4 \cdot 5H_2O$	3.0 μM
$Na_2MoO_4 \cdot 2H_2O$	1.5 μM
$CoCl_2$	1.0 μM
Vitamin B_{12}	0.1 μM

Table S2 Quantification of carotenoids and chlorophylls in *D. salina*.

Compound	Content in cells (mg/gDCW)							
	Red LED light condition					ALE		
	85	128	170	255	128(N-)	Cycle 0	Cycle 8	Cycle 16
Zeaxanthin	0.15±0.03	0.09±0.03	0.22±0.02	0.45±0.06	0.22±0.02	0.14	0.36±0.04	0.69±0.15
Lutein	4.4±0.6	5.5±0.8	4.0±0.4	2.7±0.3	8.0±0.7	3.4±0.8	5.3±0.9	7.9±0.7
Chlorophyll *b*	7.0±0.4	8.8±0.9	5.8±0.4	2.2±0.2	12.3±0.2	8.2±1.0	13.0±0.9	14.7±1.8
Chlorophyll *a*	44.2±2.3	66.4±6.9	50.8±3.0	20.5±1.4	84.3±3.9	60.5±4.9	106.0±5.3	121.8±6.2
Lycopene	0.05±0.01	0.11±0.04	0.13±0.02	0.05±0.01	0.16±0.01	0.19±0.04	0.31±0.03	0.27±0.01
All-*trans* β-carotene	1.9±0.1	4.4±0.5	2.6±0.2	1.4±0.3	7.4±0.3	2.9±0.3	7.9±1.8	9.5±0.5

Table S3 *D. salina* growth under two different light conditions [a].

Light sources	Light intensity ($\mu E/m^2/s$)	Average growth rate (gDCW/L/day)	Average biomass yield (gDCW/E)
Red LED	170	0.22±0.01	0.15±0.01
Blue and red LED (1:3)	170[b]	0.40±0.01	0.27±0.01

[a] Growth data were averaged from three independent experiments of batch culture (means±SD).

[b] The photon fluxes or light intensities were set at the same level in two conditions but the energy provided by combined blue and red LED was higher than that by red LED due to the higher energy (frequency) of blue light.

References

Chang RL, Ghamsari L, Manichaikul A, Hom EFY, Balaji S, Fu W, Shen Y, Hao T, Palsson BØ, Salehi-Ashtiani K, Papin JA (2011) Metabolic network reconstruction of *Chlamydomonas* offers insight into light-driven algal metabolism. Mol Sys Biol 7: 518

Kanehisa M, Goto S, Sato Y, Furumichi M, Tanabe M (2012) KEGG for integration and interpretation of large-scale molecular datasets. Nucleic Acids Res 40: D109–D114
